# Oxidative Stress in Poultry: Lessons from the Viral Infections

**DOI:** 10.1155/2018/5123147

**Published:** 2018-12-10

**Authors:** Zaib Ur Rehman, Chunchun Meng, Yingjie Sun, Anum Safdar, Riaz Hussain Pasha, Muhammad Munir, Chan Ding

**Affiliations:** ^1^Shanghai Veterinary Research Institute (SHVRI), Chinese Academy of Agricultural Sciences (CAAS), Shanghai 200241, China; ^2^Department of Poultry Science, Faculty of Veterinary and Animal Sciences, PMAS Arid Agriculture University, Rawalpindi 46300, Pakistan; ^3^Department of Veterinary Biomedical Sciences, Faculty of Veterinary and Animal Sciences, PMAS Arid Agriculture University, Rawalpindi 46300, Pakistan; ^4^Biomedical and Life Sciences, Lancaster University, Lancaster LA1 4YG, UK; ^5^Jiangsu Co-Innovation Center for Prevention and Control of Important Animal Infectious Diseases and Zoonoses, Yangzhou 225009, China; ^6^Shanghai Key Laboratory of Veterinary Biotechnology, Shanghai 200241, China

## Abstract

Reactive species (RS), generally known as reactive oxygen species (ROS) and reactive nitrogen species (RNS), are produced during regular metabolism in the host and are required for many cellular processes such as cytokine transcription, immunomodulation, ion transport, and apoptosis. Intriguingly, both RNS and ROS are commonly triggered by the pathogenic viruses and are famous for their dual roles in the clearance of viruses and pathological implications. Uncontrolled production of reactive species results in oxidative stress and causes damage in proteins, lipids, DNA, and cellular structures. In this review, we describe the production of RS, their detoxification by a cellular antioxidant system, and how these RS damage the proteins, lipids, and DNA. Given the widespread importance of RS in avian viral diseases, oxidative stress pathways are of utmost importance for targeted therapeutics. Therefore, a special focus is provided on avian virus-mediated oxidative stresses. Finally, future research perspectives are discussed on the exploitation of these pathways to treat viral diseases of poultry.

## 1. Introduction

The theory of oxidative stress (oxygen-free radicals) existed since the last 60 years. However, extensive research in the last three decades has clarified myriads of misconceptions and explored leading roles of oxidative stress in the pathogenesis of many viral diseases [1, 2]. A wide range of the reactive species (RS) is produced as a result of the metabolic process in the body. These RS can be reactive oxygen species (ROS) or reactive nitrogen species (RNS). Previously, RS were only considered to be toxic compounds: however, recent studies have highlighted their involvements in complex cellular signaling pathways and have improved their importance in several biological systems [3].

The ROS play vital roles in the signaling pathways, cytokine transcription, immunomodulation, ion transport, and apoptosis [4, 5]. Production of the ROS from activated innate immune cells such as neutrophils and macrophages is involved in the destruction of microbes/viruses and infected cells by oxidative bursts [6]. These ROS guide the development of adoptive immune responses, including the proliferation of T cells and positive mediation of B cell functions [7, 8].

Importantly, due to the availability of high-tech facilities, commercial poultry is reared in extensive production systems and therefore is under constant threats to pathogens including viruses [9]. These viruses can infect primarily healthy birds and occasionally vaccinated flocks and cause an irreversible damage to different body tissues. Several viral diseases affect the production of the ROS [10–12], and overproduction of ROS may cause the damage to DNA, protein, and lipid structures [13], leading to the disruption of the cell functions. This imbalance in the production and detoxification of the ROS is collectively referred as oxidative stress. This review aims at highlighting the molecular mechanisms of oxidative stresses, deleterious effects on cell functions, and their roles in the pathobiology of avian viral infections.

## 2. Reactive Species, Oxidative Stress, and Antioxidant System

**Figure 1:**
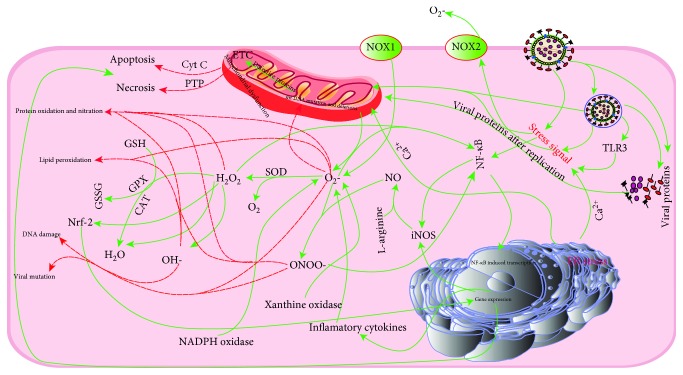
Basic mechanisms of viral cross-talk with the cellular pathways to cause oxidative damage to cellular components. After entry into the cells, viral particles like proteins or nucleic acids are recognised by the pattern recognition receptors. Viral recognition as well as replication initiates the stress signalling and sends signal to the mitochondria and NOX2 and activates the NF-*κ*B. After receiving the stress signals, NOX2 initiates the production of superoxides (O_2_^−^), and dysfunctioning in the mitochondrial proteins function occurs. These defective mitochondrial proteins result in the leakage of electrons and superoxides from the mitochondria, as well as initiating the cell death pathways by cytochrome c (cyt c) or permeability transition pore (PTP). The NF-*κ*B-induced transcription is initiated by the NF-*κ*B resulting in the production of many cytokines as well as inducible NO synthase (iNOS). This iNOS produces large amounts of nitric oxide (NO). The NO and O_2_^−^ react together to produce peroxynitrite (ONOO) which is a highly reactive compound and can cause the protein nitration, lipid peroxidation, DNA damage, and viral mutations. Similarly, higher production of O_2_^−^ results in the production of H_2_O_2_ by the catalytic activity of superoxide dismutase (SOD). Uncontrolled production of H_2_O_2_ produces hydroxyl radicals (OH-) via reaction with metal cations, and these H_2_O_2_ and OH- cause irreversible damage to cellular macromolecules: proteins, lipids, nucleic acids, etc.

**Scheme 1:**
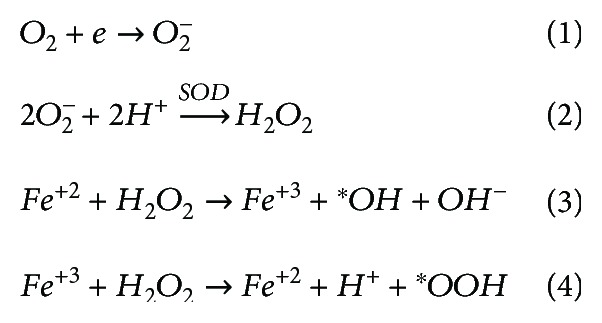
Production of ROS and Fenton reaction.

Owing to the production-dependent oxidative stresses, exploring the molecular mechanisms of ROS production in living organisms is imperative. ROS are primarily produced from the mitochondria, endoplasmic reticulum, plasma membrane, and peroxisomes [14, 15]. Since most of the oxidative processes take place in the mitochondria in an effort to generate energy (about 18 times more energy is produced from oxidative process than from the conventional glycolysis [16]), more than 90% of total ROS in eukaryotes is produced by the mitochondria [17]. In the living organisms, most of the consumed oxygen is converted to water in the electron-transport chain (ETC) by the cytochrome c oxidase without any contribution to ROS production [18]. These ROS include superoxide anion (O_2_^−^), hydroxyl radical (OH), hydrogen peroxide (H_2_O_2_), hydroperoxyl (HO_2_), and hypochlorous acid (HOCl). Although all these ROS are important, the OH is of utmost importance due to its high reactivity, high mobility, low-molecular weight, and water solubility (Figure 1). A cell produces 50 hydroxyl radicals every second, which are about 4 million hydroxyl radicals in a day [19]. These radicals are generally neutralized and in worse cases could attack the cellular biomolecules leading to many diseases such as neurodegeneration, cardiovascular disease, and cancer [14]. Electrons from the ETC can be transferred to the O_2_ resulting in O_2_^−^ (Scheme 1, reaction (1)) by the process of oxidative phosphorylation. Another mode of O_2_^−^ production is through the degradation of purine nucleotides to xanthine and hypoxanthine and subsequently to uric acid via xanthine oxidase (XO) [20, 21]. Hypoxic condition activates the XO by the posttranslational modification of xanthine dehydrogenase (XD). These changes lead to the excessive production of O_2_^−^ and H_2_O_2_.

In avian diseases, pathogens are recognised by the innate immune system leading to the production of O_2_^−^ in the phagosome and outside the cells by the process of oxidative or respiratory burst, catalysed by the NADPH oxidase complex (NOX). This process of ROS production is critical to promote cellular responses [7]. The O_2_^−^, produced by the immune cells, can lead to the formation of other ROS such as HOCl, H_2_O_2_, peroxynitrite (ONOO^−^), and OH [20, 22]. The OH radicals are produced from O_2_^−^ by Fenton reaction (Scheme 1: reactions (2)–(4)). Another possible mechanism is the triggering of ROS production by the virus-induced cytokines. Taken together, ROS may be generated from the activation of XO, NADPH oxidase, lipoxygenases, and cyclooxygenase or from the leakage of electrons from ETC [23].

The RNS are different products, derived from nitric oxide (NO), including nitrogen dioxide (NO_2_), dinitrogen trioxide (N_2_O_3_), nitroxyl anion (HNO), nitrosonium (NO^+^), nitronium (NO_2_^+^), ONOO^−^, nitrousoxide (HNO_2_), nitrosoperoxycarbonate anion (ONOOCO_2_), S-nitrosothiols (RSNOs), nitryl chloride (Cl-NO_2_), and alkyl peroxynitrates (RONOO) [24]. The RNS are produced mainly from the NO, which is produced by the NO synthases (NOS), from L-arginine and oxygen. Direct biological action of NO is limited due to its less movement ability and less biological half-life *in vivo*. The NO_2_ and NO_3_ are considered to be the final products of NO and are produced by the oxidation of NOS-derived NO. Similarly, another RNS, ONOO^−^, is a result of the reaction of NO and O_2_^−^. This ONOO^−^ reacts with tyrosine residues to result in nitration and reacts with CO_2_ leading to the formation of carbonate (CO_3_^−^) and NO_2_. Peroxynitrite affects many biological molecules (Figure 1) such as modification of receptors [25–27], calcium dysregulation [28, 29], mitochondrial dysfunction [30, 31], nitration and peroxidation of lipids [32], protein damage [33], and DNA damage (Figure 1) [34]. These NO_2_ and CO_3_^−^ have strong ability to nitrate the proteins, lipids, and nucleic acids.

To cope with the oxidative stress, induced by the overproduction of the above-mentioned ROS and RNS, birds have a multilayered and well-defined antioxidant system. It is comprised of an enzymatic antioxidant system made up of catalases (CAT), superoxide dismutase (SOD), glutathione peroxidase (GPx), and glutaredoxins (GR) and a nonenzymatic system which is composed of glutathione (GSH), vitamin E, vitamin C, carotenoids, flavonoids, anserine, carnosine, homocarnosine, and melatonin. Enzymatic antioxidants are always produced in the body; however, they require cofactors such as zinc, magnesium, copper, manganese, iron, and selenium for their optimal functions whereas nonenzymatic antioxidants are naturally produced in situ or supplied by food/feeding [11]. Therefore, dietary supplementation of antioxidants may be a promising factor to reduce the damage caused by virus-induced oxidative stresses in the poultry [5].

To prevent the oxidative damage to cells/tissues, O_2_^−^ is converted to H_2_O_2_ by the enzymatic action of SOD. There are four different selenium-dependent forms of GSH-Px in birds, which primarily convert the hydroperoxides and H_2_O_2_, to the H_2_O and O_2_ by using the GSH [35], whereas CAT also perform the same function (Figure 1).

## 3. Consequences of Oxidative Stress

Due to continued production, all living organisms are in a constant struggle to minimize the oxidative damage. Excessive production of ROS and RNS has been observed in many viral infections [5, 10, 11, 20, 36]. Oxidative stress conditions contribute to the pathogenesis of viral infection. Even though these ROS and RNS are involved in many signalling pathways in viral diseases, the imbalance of ROS and RNS production and poor detoxification lead to extensive damage to many cellular compounds such as lipids, nucleic acids, and proteins (Figure 1).

### 3.1. Nucleic Acid Damage

All the organic molecules are susceptible to oxidative stress; however, the most important impact is nucleic acid damage [37]. Oxidative stress-induced DNA damage may result in genomic instability, modification of nitrogenous bases and/or sugars, double-stranded DNA breaks, translocation, increased mutation rates, and apoptosis [34, 38–40]. Virus-induced oxidative stress directly or indirectly causes the DNA damage by modifying the nucleobases and sugar backbone and results in strand crosslinking, breakages, and base loss. Reactive species such as O_2_^−^, H_2_O_2_, and HO_2_ lack any marked reactivity to nucleobases and 2-deoxyribose, but OH reacts with DNA in different ways. Recently, excellent reviews have been published on the oxidative damage to DNA [16, 40, 41]. Briefly, the production of OH radicals from the O_2_^−^ by Fenton-type reaction reacts with the double bond of the 5,6-pyrimidine and 7,8-purine nucleobases leading to the formation of radical intermediates, which may react as oxidising agents [42]. Another most common method is the abstraction of hydrogen from the thymine and 5-methylcytosine by OH radical, resulting in the formation of 5-(uracilyl) and 5-(cytosyl) methyl radicals [43]. These abstractions of hydrogen atoms at C3 and C5 result in the strand breakage; however, the abstraction of hydrogen at C4 results in more complex reactions. Guanine *moiety* most frequently undergoes oxidation by the RNS and ROS due to its lower reduction rate. Oxidation of adenine may be the initial site; however, it is repaired by the neighbouring guanine leading to the production of highly mutagenic 8-hydroxyguanine.

Among the RNS, ONOO^−^ and NO_2_ are the most important in causing nucleic damage. The ONOO^−^ reacts with guanine nucleobases to form 8-nitroguanosine, 8-nitroguanine, 8-nitrodeoxyguanosine, and 8-oxodeoxyguanine. The 8-nitroguanine induces the transversion of G:C to T:A in the DNA [44]. Likewise, 8-nitroguanosine is a highly reactive nucleic acid derivative, which uncouples NADPH electron transport through the cytochrome-NOS complex leading to the production of O_2_^−^ [45]. Furthermore, 8-nitrodeoxyguanosine may be incorporated into the DNA by thymine or adenine, resulting in mutation and protein alteration. Proliferating cells are highly prone to nucleic acid damage by ROS and RNS, because those cells have dissociated histone from DNA which cannot protect them from the oxidative damage [20]. These reactive species also increase the mutation rate in viruses, particularly RNA viruses [46]. One of the most common damages by virus-induced oxidative stresses occurs to mitochondrial DNA (mDNA) due to ineffective repair mechanisms. ROS and RNS react with mDNA leading to mitochondrial dysfunction and activation of different cell death pathways (Figure 1).

### 3.2. Protein Damage

Extensive research has been conducted on the oxidative modification of proteins. These are the main targets of oxidants within the cell (about 69%) compared to lipids and nucleic acids (about 18% and 15%, respectively) [47, 48]. ROS and RNS react with proteins, resulting in the fragmentation of peptide chain, decreased protein solubility, aldehyde and ketone production, crosslinking of proteins, and oxidation of specific amino acid [20, 39, 49, 50]. Oxidative stresses can affect the proteins in a variety of ways both directly or indirectly. Direct oxidation is performed by different ROS, and indirect modification is mediated by oxidized forms of lipids and carbohydrates. Examples of direct modification include carbonylation, nitrosylation, glutathionylation, and disulphide bond formation of proteins. The second way of protein modification is through the oxidative products of lipids, proteins/amino acids, carbohydrates, and glutathione [51, 52]; i.e., lipid peroxidation products from the hydroxynonenal, malondialdehyde, and acrolein react with proteins to induce protein oxidation [53, 54].

Different amino acids in the polypeptide chain differ in their susceptibility to oxidative stress. Sulphur-containing amino acids and thiol groups are more susceptible to oxidative stress [51]. ROS removes the hydrogen atom from the cysteine residue leading to the formation of thiyl radical, which reacts with the second thiyl radical to form disulphide bond and sulfenic, sulfinic, and sulfonic acids. Another way of oxidative damage is the addition of oxygen to methionine residue resulting in the formation of methionine sulphoxide derivative [55]. Tyrosine oxidation and nitration are mediated by O_2_^−^, ONOO^−^, and NO_2_ to form bityrosine and 3-nitrotyrosine (markers of nitrative stress) [56–58]. These oxidised proteins undergo proteolytic digestion and proteasomal degradation. The 3-nitrotyrosine severely affects the microtubule structure leading to the functional impairments in the cell. The O_2_^−^, ONOO^−^, H_2_O_2_, and NO irreversibly react with iron-sulphur centres of metalloproteins and result in the inactivation of the enzymes [20, 57, 59, 60]. Reactive species including ONOO^−^ also inactivate the inhibiters of the matrix metalloproteinases and *α*-1 proteinase [59, 61], ultimately causing more tissue damage in viral infections. ROS and RNS also enhance the inflammatory response, mitochondrial damage, and cytochrome c release and result in apoptosis and necrosis [59, 62, 63].

### 3.3. Lipid Damage

Lipids are comparatively reduced molecules and an important cellular component [64]. Lipids undergo oxidation in the presence of ROS and/or RNS [65] and have been associated with the pathophysiology of many diseases. Oxidation of lipids is a complex process which is influenced by different factors including the degree of unsaturated fatty acids, position of fatty acids in the triacylglycerol molecules, lipid class, and presence of antioxidants in lipids [66]. Oxidation and nitrosylation of lipids generate highly reactive electrophilic aldehyde, peroxide adducts, and ketones. These molecules disrupt the lipid bilayer, cause inactivation of enzymes and other cellular proteins and membrane-bound receptors, and increase tissue permeability and diffusion [39, 67, 68]. The oxidation process of lipids is catalysed by different enzymes like lipoxygenases, cyclooxygenases, and cytochrome P450 [69]. Polyunsaturated fatty acids and low-density lipoprotein are the major targets of oxidation leading to cellular and tissue damages. For example, oxidation of lipids with ROS produces aldehydes, which react with proteins, nucleic acids, and other hydrocarbons.

**Scheme 2:**
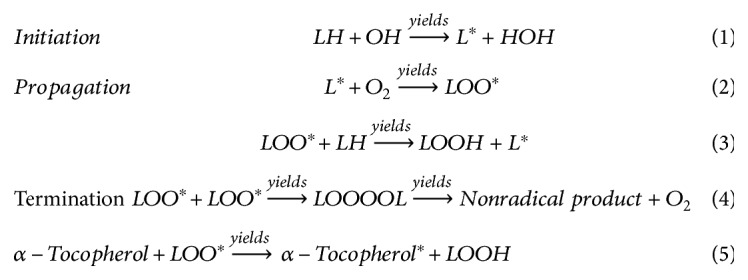
Lipid peroxidation mechanism.

Lipid peroxidation is a three-step process, consisting of initiation, propagation, and termination [14, 69]. Initiation of oxidation can be mediated by different stimuli including gamma irradiation, transition metals, enzymes, hydroxyl radicals, and pathogen stress. These initiators like OH react with unsaturated lipids (LH) and extract the allylic hydrogen from lipids to produce alkyl radical (L^∗^) of unsaturated fatty acid (Scheme 2: equation (1)). In the propagation step, O_2_ reacts with L^∗^ to form lipid peroxy radical (LOO^∗^) (Scheme 2: equation (2)). Then, LOO^∗^ reacts with another unsaturated lipid (LH) to form hydroperoxides and lipid radical (L^∗^) (Scheme 2: equation (3)). In the last stage of lipid peroxidation, two LOO^∗^ react with each other to form a nonradical product. Many antioxidants, like vitamin E, can dismiss the propagation step of lipid peroxidation. Vitamin E works as a chain-breaking antioxidant, reacts with LOO^∗^ to donate hydrogen ion, and converts to vitamin E radical and lipid hydroperoxide (LOOH) (Scheme 2: equation (5)). Vitamin E radical can be converted to nonradical vitamin E in the subsequent reaction by the ascorbic acid (vitamin C) or glutathione. The LOOH can decompose to generate different lipid peroxidation products; however, among those, malondialdehyde (MDA), 4-hydroxynonenal (4-HNE), hexanal, and propanal are the most studied [14, 70–74]. Comprehensive reviews and book chapters with chemistry detail of every step are available [66, 69, 75].

NO is not a strong oxidant and cannot directly abstract the bis-allylic hydrogen from fatty acids to initiate the lipid peroxidation [76], but its products such as NO_2_ and ONOO^−^ initiate lipid oxidation [77]. In fact, NO is an inhibitor of lipid oxidation by facile scavenging of lipid peroxyl radicals [76, 78].

## 4. Avian Virus-Induced Oxidative Stress and Antioxidants

Innate immune cells are activated in all the viral infections, causing the production of ROS and prooxidant cytokines and enhancing the iron uptake of a mononuclear phagocytic system (reticuloendothelial system) [79]. Viruses enhance the production of oxidants such as superoxide and NO and prevent the synthesis of CAT, SOD, and GPx resulting in the disruption of the redox balance. Less production and activity of these enzymes lead to a weak immune response, as these are required in high quantities for immune cells compared to other cells [11].

During viral infections, production of ROS is increased from the granulocytes and macrophages and exerts antimicrobial action against many pathogens [6]. Failure to ROS production leads to many opportunistic pathogens including *Salmonella*, *Staphylococcus aureus*, *Serratia marcescens*, and *Aspergillus* spp. [80–83]. The direct antimicrobial action includes oxidation of DNA, protein, and lipid peroxidation [84]. Upon viral infection, ROS triggers a different pathway to kill or spread viral infections, including autophagy [85], apoptosis [86], and inhibition of mammalian target of rapamycin [87]. Moreover, ROS also interfere with the antigen presentation by innate immune cells, T cell polarization, and adaptive immune responses [84]. At the same time, research also supports the immunosuppressive effects of ROS which may also facilitate the viral infection and evolution [88].

In the following sections, a disease/virus-wise cellular senescence in poultry is discussed.

### 4.1. Newcastle Disease Virus

*Avian avulavirus 1*, also known as Newcastle disease virus (NDV), is one of the most important pathogens affecting the poultry industry worldwide [89]. The first evidence of virus-induced oxidative stress came from the paramyxovirus, where it was highlighted that Sendai virus induces the oxidative stress by increasing the production of RS [90].

Mesogenic and velogenic NDV cause the oxidative stress and increase the level of MDA and decrease the GSH and activities of SOD, CAT, GPx, GR, and GST in the brain and liver of chickens [91, 92]. Similarly, increased concentrations of the NO and MDA were noted in NDV-infected chickens [93]. NDV also increases the XO, uric acid (UA), superoxides, intracellular protein carbonyls (PCO), and nitrates in the brain and liver of infected birds [92, 94]. These adverse oxidative effects created by the NDV can be mitigated by the supplementation of vitamin E (Table 1) [92, 94]. It has been reported that haemagglutinin-neuraminidase (HN) increases the oxidative stress in chicken embryo fibroblast [95]. Further studies are needed to determine the role of other viral proteins and patterns of the oxidative stress in different tissues, which are mainly affected in Newcastle disease. Many studies have demonstrated the increased level or expression of NO in NDV-infected birds or cell lines [93, 96–101]. These increased concentrations of RS are associated with the tissue damage in the brain and intestine of chickens [91]. Recently, saponins have shown the immune stimulatory for NDV [102, 103] and antioxidant properties for cyclophosphamide-induced oxidative stress in chicken [104].

### 4.2. Avian Influenza Virus

Avian influenza is the most serious zoonotic disease, caused by avian influenza virus (AIV), affecting the poultry industry worldwide. Extensive efforts to explore the pathology of AIV have revealed that RS plays an important role in mammals. But studies related to the role of oxidative stress induced by AIV are less in birds. AIV infection induces a strong influx of inflammatory cells, leading to the increased production of ROS by activating NADPH oxidase activity. Ye et al. [105] have performed a comprehensive study to elucidate the role of oxidative stress in the pathogenicity of H5N1, H7N9, H5N3, and H1N1 in different cells like adenocarcinomic human alveolar basal epithelial cells (A549), Madin-Darby canine kidney (MDCK) cells, chicken HD-11 macrophage, and DF-1 embryo fibroblast. Results indicate that inhibition of a Nox2 by apocynin inhibits the production of cytokines and reactive oxygen species (Table 1). Apocynin has also increased the virus-induced mRNA and protein expression of SOCS1 and SOCS3, which enhance the negative regulation of cytokines. In another study, Qi et al. [36] have found that NS1 protein of the H9N2 AIV is responsible for the ROS production and oxidative stress in primary chicken oviduct epithelial cells (COECs) (Table 1). This disturbance of cellular redox homeostatic causes the apoptosis of COECs via a mitochondria-dependent pathway. NO is involved in the pathogenesis of influenza, and results of the previous studies indicate that inhibition of NO production increases the survival rate in influenza [106, 107]. Increased concentration of NO and/or iNOS expression was observed in influenza-infected chickens and ducks [108–110]. A number of studies suggest that RS increases the mortality, lung injury, and inflammation in influenza infection [111, 112]. Administration of antioxidants including vitamin E, vitamin C, N-acetyl-L-cysteine, pyrrolidine dithiocarbamate, glutathione, resveratrol, ambroxol, isoquercetin, and quercetin decreases the pathological effects caused by the influenza virus [113–116].

### 4.3. Avian Reovirus

Avian reoviruses (ARV) are the members of Orthoreovirus genus which belongs to the Reoviridae family. ARV is a pathogenic agent for chicken, turkeys, ducks, geese, and many other species of birds and causes viral arthritis/tenosynovitis, stunting syndrome, respiratory and enteric disease, immunosuppression, and malabsorption syndrome [117, 118]. The ARV and its *σ*C protein have been shown to increase the lipid peroxidation and generation of ROS (Table 1). Furthermore, ARV and *σ*C also induce DNA damage which was confirmed by comet assay and expression patterns of DNA-damage-responsive gene DDIT-3 and H2AX phosphorylation [119]. This DNA damage response might be associated with the ROS because DDIT-3 has been shown to be induced by ROS [120]. Overexpression of DDIT-3 may be the reason for ARV-induced apoptosis because it has been confirmed in many other viral infections [121, 122].

### 4.4. Duck Hepatitis Virus

**Figure 2:**
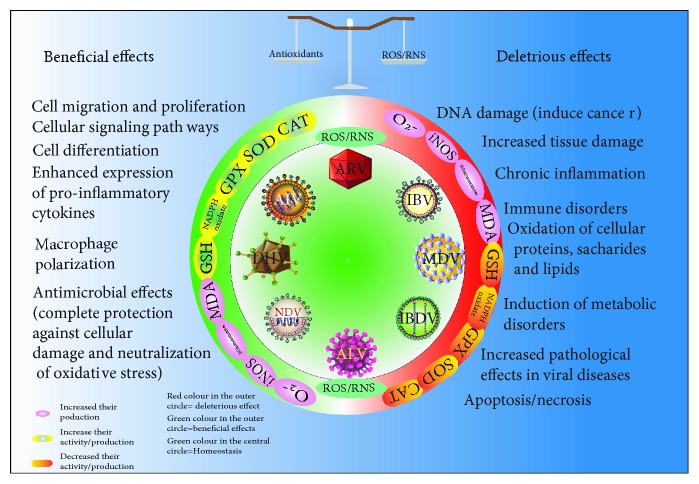
The scheme summarizes the effect of common avian viruses on the production of reactive oxygen species (ROS) and reactive nitrogen species (RNS). After viral insult, cells recognise them by different pattern recognition receptors and enhance the production of ROS/RNS species, which are involved in the cell migration, cell signalling, macrophage polarization, requirement of immune cells, and importantly clearance to host from invading pathogens. But in chronic or overproduction of viruses, hijack the production of ROS/RNS by disturbing different cellular pathways/organelles like mitochondrial metabolism, leading to a decrease the activity/level of cellular enzymatic and nonenzymatic antioxidants. It leads to increased pathological damage in poultry. It leads to increased pathological damage in poultry.

Duck virus hepatitis caused by duck hepatitis A virus (DHAV) is an acute, contagious, and lethal disease of young ducklings, characterized by rapid transmission and severe hepatitis, which was first described on Long Island, NY, USA, in 1949 [123–125]. There are three different serotypes (1, 2, and 3) of DHAV; from these, serotype 1 (DHAV 1) is commonly distributed and the most virulent compared to others [126]. DHAV leads to persistent infection and causes oxidative stress in ducks. Culturing of LMH chicken hepatoma cells in the presence of different concentrations of the hydrogen peroxide increases the integration of the duck hepatitis virus (DHV) genome into the host genome in a dose-dependent way [127]. DHAV 1-infected ducklings show higher plasma levels of iNOS and MDA and decreased level of the GPx and CAT (Table 1), which leads to necrosis as well as apoptosis of hepatocytes [128]. Supplementation of icariin, phosphorylated icariin, and baicalin-linarin-icariin-notoginsenoside R1 (BLIN) decreases the hepatocyte damage caused by DHAV by attenuating the oxidative stress [123, 128]. Another study confirmed that *Taraxacum mongolicum* extract protects the duck embryo hepatocytes from the infection of duck hepatitis B virus by alleviating the oxidative stress [129]. Furthermore, these studies confirm that DHAV causes damage to hepatocytes by oxidative stress, and prevention of oxidative stress lessens the tissue damage, necrosis, and mortality of duckling, clearly indicating the role of oxidative stress in the pathogenesis of DHV (Figure 2).

### 4.5. Infectious Bronchitis Virus

Infectious bronchitis virus (IBV) causes infectious bronchitis in poultry and is endemic in all poultry-producing regions of the world. The IBV virulence affects the oxidative status by differentially modulating MnSOD. Highly virulent strain significantly increases the level of MnSOD than an attenuated virus. Increased level of MnSOD may direct the more significant immune response to eradicate the virus [130]. The same group of researchers also demonstrated that IBV infection increases the abundance of glutathione S-transferase 2, a protein of the sulfotransferase family, and L-lactate dehydrogenase [131].

### 4.6. Infectious Bursal Disease Virus

Infectious bursal disease (IBD) or Gumboro is caused by the infectious bursal disease virus (IBDV), which is one of the most devastating diseases of poultry, worldwide [132]. In intensive poultry production, IBD causes heavy economic loses by causing 80–100% mortality and prolonged immunosuppression [133]. The primary replication site of IBDV is the bursa of Fabricius, where sever destruction of the B lymphocytes causes significant impairment of the antibody response [132]. Infectious bursal disease virus (IBDV) infection of bursal lymphocytes increases intracellular ROS levels, decreases the GSH content and activities of GPx and SOD [134], and increases serum levels of lipid peroxidation (Table 1) [135]. The increased level of ROS may be involved in the shutoff cellular protein synthesis [136], because ROS are involved in the activation of the protein kinase R pathway [137], leading to cell death [136, 138]. Although the precise pathway of IBDV-induced apoptosis is not known, it has been shown that overexpression of oral cancer overexpressed 1 (ORAOV1) protein decreased the release of IBDV from infected cells. Stable overexpression of ORAOV1 is involved in the resistance to oxidative stress [139] which may decrease the IBDV-induced apoptosis [140]. These IBDV-induced oxidative stress and mortality can be reduced by Sargassum polysaccharide, Ginsenoside Rg1, and vitamin E supplementation (Table 1) [134, 135, 141, 142].

### 4.7. Marek's Disease Virus

Marek's disease (MD), caused by the MD virus (MDV) also known as *Gallid herpesvirus 2*, is an important neoplastic disease of poultry. MD has been shown to cause the aberrations in the oxidative status of birds (Table 1). Hao et al. [143] have found that MDV infection of chickens leads to increased lipid peroxidation and decreased activity of Se-GSH-PX in the spleen, thymus, bursa, heart, liver, kidneys, and gonads. Similarly, Kishore [144] found the decreased activities of SOD, CAT, GST, and GPX and level of GSH in the liver of MDV-infected chickens. Keles et al. [145] have demonstrated that MD induces DNA damage and increases concentration of MDA and PCO and plasma concentration of NO. Furthermore, it also decreases the total antioxidant activities as well as GSH in MDV-infected birds. The MDV-infected chicken shows a significant positive correlation between DNA damage, MDA, PCO, and NOx. Results of Bencherit et al. [146] suggest that MDV infection increases the production of ROS and RNS and induces DNA damage. This DNA damage may be the result of ROS and RNS. MDV infection only causes the DNA breaks in lytically infected cells and not in latently infected cells. DNA damage in MDV-infected cells is caused by the viral protein 22 and might be involved in the oncogenicity of MDV [146].

### 4.8. Avian Leukosis Virus

Avian leukosis virus subgroup J (ALV-J) is an oncogenic virus, belongs to genus *Alpharetrovirus* of the subfamily *Orthoretrovirinae* of family *Retroviridae*, and causes immunosuppressive and oncogenic disease in poultry, leading to heavy economic losses [147–149].

The ALV-J induces the production of NO from monocyte-derived macrophages at 12, 24, and 36 hours postinfection [150], but this production was not too much. However, the results of Landman et al. [151] demonstrate the nonsignificant effect of ALV-J on NO of spleen-derived macrophages. Birds infected with avian erythroblastosis virus show suppressed splenic T cell mitogen responses [152]. These immune dysfunctions can be ameliorated by the supplementation of vitamin E, Trolox, butylated hydroxyanisole, and butylated hydroxytoluene [153]. These protective effects of antioxidants indicate the involvement of oxidative stress in retrovirus infection in chicken. Another indirect evidence indicates the involvement of oxidative stress in avian sarcoma and leukosis virus infection, as it increases the cellular DNA damage response in infected cells [154].

## 5. Conclusion

Production of RS by the innate immune cells is a typical process in viral diseases to counteract their replication. Nonetheless, many viruses employ different strategies to manipulate this phenomenon and it became overwhelming for the endogenous antioxidants leading to the oxidation of lipids, proteins, nucleic acids, cell membranes, and other organelles. Scavenging of oxidative stress is an important tool to prevent tissue damage and severe complications associated with the viral diseases in the poultry. Many antioxidants have been proven to prevent the oxidative stresses, enhance the immune responses, and inhibit the virus replication, which can be used to decrease the tissue damage and complications associated with viral diseases in poultry. It would be of great interest to supplement the antioxidants such as vitamin E, vitamin C, N-acetyl-L-cysteine, pyrrolidine dithiocarbamate, glutathione, resveratrol, ambroxol, isoquercetin, and quercetin to decrease the pathological effects triggered by avian viral diseases. However, clinical trials are required to demonstrate the therapeutic roles of these antioxidants in avian viral diseases.

## Figures and Tables

**Table 1:** Effect of avian viral infections on the oxidative stress parameters.

Purpose of study	Virus/viral protein	Animal/cell line	Oxidative result	Other important results	Reference
ARV-mediated apoptosis	ARV and its encoded protein σC	DF1 cell	Increased ROS and lipid peroxidation leads to DNA damage		[119]
Effects of different concentrations of hydrogen peroxide on the frequency of hepadnaviral DNA integrations	Duck hepatitis B virus	Chicken LMH-D2 cell line	Increase viral DNA integrations in host DNA in a dose-dependent manner		[127]
Antioxidant effects of vitamin E on the liver, brain, and heart of Newcastle disease virus- (NDV-) infected chickens	Mesogenic NDV	Chicken	NDV infection increases in MDA levels and decreases activities of SOD, CAT, GPx, GR, GST, and levels of GSH in the brain and liver vitamin E lessens these effects	NDV induces histological changes in the brain, liver, and heart	[92]
Investigate the role of NDV-induced oxidative stress in pathogenesis and protective effects of vitamin E	Mesogenic NDV	Chicken	NDV infection increases XOD activity, UA, and superoxide radical level as well as intracellular protein carbonyls and nitrates in the brain and liver. Vitamin E mitigates NDV-induced oxidative damage	NDV increases the apoptosis in the brain	[94]
Effect of NDV-induced pathological changes in the brain and protective effects of vitamin E	ZJ1 (velogenic NDV)	Chicken	ZJ1 infection causes increased concentrations of MDA and NO and decreased level of TAOC and GSH, along with decreased activities of CAT, SOD, and GPx in the brain and plasma	Vitamin E supplementation lessens the oxidative stress and histopathological changes in the brain	[91]
To study the nature and dynamics of NDV-induced oxidative stresses in the intestine of chickens	ZJ1 (velogenic NDV)	Chicken	Virulent NDV infection leads to increased concentrations of MDA and NO and decreased level of TAOC and GSH, along with decreased activities of CAT, SOD, and GPx in the duodenum and jejunum	Oxidative stress and tissue damage in the duodenum and jejunum can be minimized by supplementation of vitamin E	[5]
How haemagglutinin-neuraminidase (HN) protein causes apoptosis?	Newcastle disease virus, HN protein	CEF cells	Increased fluorescent intensity from dichlorofluorescin diacetate from HN-infected cells	Oxidative stress may be the cause of apoptosis	[155]
Role of oxidative stress in the pathogenesis of Duck viral hepatitis and protective role of icariin or p-icariin	Duck hepatitis virus 1	Ducklings	DHV-1 induced significant oxidative damage in ducklings	Icariin or p-icariin attenuated liver pathological injury and attenuates oxidative stress	[128]
Baicalin-linarin-icariin-notoginsenoside R1 protective effects in DHV-induced injury	Duck hepatitis A virus 1	Ducklings	BLIN alleviates the oxidative stress	BLIN showed a significant curative effect on DVH	[123]
To validate the antiviral effect of *Taraxacum mongolicum* extract (TME)	Duck hepatitis B virus	Duck embryo hepatocytes	Protect hepatocytes by ameliorating oxidative stress	Antiviral effect of TME may contribute to blocking protein synthesis steps and DNA replication	[129]
To examine the proteome profiles of tracheal and kidney tissues from chicken infected with highly virulent and attenuated IBV	Highly virulent and attenuated IBV	Chicken	Virulent virus increasing the MnSOD protein than attenuating	Some proteins involved in cytoskeleton organization and stress showed changes according to virus strain	[130]
To determine the antioxidant effects of *Sargassum* polysaccharide in IBDV induces oxidative stress	IBDV	Lymphocytes	IBDV infection increases intracellular ROS levels, decreases in GSH content, and decreases activities of GSH-Px and SOD	*Sargassum* polysaccharide prevents the lymphocytes from oxidative stress	[134]
To examine oxidative stress and DNA damage caused by MDV	MDV	Chicken	Increase MDA and PCO and NO metabolites and decrease in antioxidant activity and GSH	Positive correlation exists between DNA damage, MDA, PCO, and NOx in MDV-infected birds	[145]
Effect of inhibition of ROS production by apocynin on host cytokine homeostasis	H1N1, H5N3, H5N1, H7N9	A549, MDCK, HD-11, and DF-1 cells	Apocynin inhibited the ROS production from infected cells	Apocynin increased the expression of SOCS1 and SOCS3 and inhibited the influenza-induced cytokines	[105]
To elucidate the role of H9N2 NS1 protein in the pathogenicity in the COECs	H9N2 NS1 protein	COECs	H9N2 NS1 protein increases the ROS production and decreases SOD activity	Pyrrolidine dithiocarbamate (PDTC) or N-acetylcysteine (NAC) significantly inhibited NS1-induced apoptosis	[36]
